# Cardiovascular Disease Research: Time to Focus on Minority Ethnic Groups

**DOI:** 10.1371/journal.pmed.0030081

**Published:** 2006-01-03

Long-standing inequities exist in the amount of research on diseases that affect the developed world and those—such as leishmaniasis and schistosomiasis—that affect nonwhite populations who live in the developing world. These inequities have also been perceived to exist when white and nonwhite populations coexist in developed countries.

Ethnic groups differ in their susceptibility to particular diseases. These differences can be genetic—the result of gene mutations that are more prevalent in some ethnic groups. They may also be due to social factors—in industrialized countries, for example, ethnic minorities are often poorer, less educated, and more frequently unemployed than their white counterparts. Studies in white populations, therefore, cannot necessarily be extrapolated to other ethnic groups.

Cardiovascular disease is a major cause of death for all ethnic groups in developed countries, and the risk is especially high in those originating from South Asia. In the UK, for example, early deaths from coronary heart disease in Indians, Bangladeshis, Pakistanis, and Sri Lankans are about 50% higher than the national average. For Caribbeans and West Africans, on the other hand, the rates are much lower than average.

Meghna Ranganathan and Raj Bhopal systematically reviewed the scientific literature over the past decades to assess the extent to which different ethnic minorities were included in cardiovascular cohort studies in Europe and North America.

They identified 72 studies, 39 of which started after 1975 (at that time, it was well-known that different ethnic groups have different risk factors, and quite a bit was known about causes and control of the disease in white populations). Forty-one studies were conducted in Europe and 31 in North America, and one study involved participants from both continents.

Overall, the researchers found, there is little information on cardiovascular research in ethnic populations—just ten studies compared white and nonwhite populations, and only five focused on one nonwhite ethnic group. All 15 of those were conducted in the United States. Despite the high risk of cardiovascular disease in ethnic minorities in Europe, not one European study so far has investigated the disease specifically in these populations.

In general, it seems that issues of race or ethnicity have rarely been taken into account. Decisions about which ethnic groups to include were not often a part of the study design nor were they made explicit in the study report, and few articles gave details of the ethnic composition of the study population. Even when nonwhite participants were included in studies, there were often too few of them to allow for analysis by ethnicity.

In some cases, researchers were open about their aim to study only white populations. In others, by selecting participants based on employment status or by conducting studies in rural settings, researchers were unlikely to include many participants from nonwhite ethnic groups that tend to cluster in cities.

In cardiovascular disease, in particular, clear ethnic variations in risk mean that studying nonwhite populations is crucial if their health needs are not to be neglected, say the authors.

The first step toward better understanding why some ethnic groups are more susceptible to disease is acknowledging the need for studying the question. Because such studies are expensive and challenging, say Ranganathan and Bhopal, they will only be done when there is a demand for them.

